# Using auxotrophic donor strains to explore pQBR57 plasmid host range among environmental soil bacterial isolates

**DOI:** 10.1099/mic.0.001737

**Published:** 2026-07-10

**Authors:** Alejandro Marquiegui-Alvaro, Anastasia Kottara, Matthew J. N. Thomas, Alberto Scarampi, Micaela Chacón, Michael Brockhurst, Neil Dixon

**Affiliations:** 1 Manchester Institute of Biotechnology (MIB), Department of Chemistry, University of Manchester, Manchester, M1 7DN, UK; 2 Division of Evolution, Infection and Genomics, School of Biological Sciences, Michael Smith Building, University of Manchester, Manchester, M13 9PT, UK; 3 School of Life Sciences, Gibbet Hill Campus, University of Warwick, CV4 7AL, UK

**Keywords:** auxotrophic plasmid donor strain, bioremediation, horizontal gene transfer, plasmid conjugation, pQBR57 plasmid, *Pseudomonas*

## Abstract

Plasmid host range (PHR) plays a key role in the spread of ecologically important genes, alongside applications in microbiome engineering and environmental biotechnology. PHR is a complex trait arising from the combination of plasmid, donor and recipient properties. Most studies of PHR use a single donor strain, leaving the role of the donor unexplored and often require genetically tagged recipient strains for counter-selection, which limits the use of non-genetically tractable strains. Here, we applied auxotrophic donor counter-selection in a relatively high-throughput and accessible screening format to characterize PHR across a diverse collection of environmental isolates without the need for recipient engineering. Specifically, we used two auxotrophic donors (*Pseudomonas fluorescens* and *Pseudomonas putida*) and plasmid pQBR57-tphKAB, an environmental plasmid engineered for terephthalic acid bioremediation. We screened a library of 101 soil isolates as potential recipients, including genera such as *Pseudomonas*, *Bacillus* and *Xanthomonas*. We only observed conjugation into other *Pseudomonas*, but donor identity was found to affect PHR, with *P. fluorescens* conjugating the plasmid into more recipient strains than *P. putida*. Phylogenomic analysis revealed that transconjugants clustered primarily with the *Pseudomonas citronellolis* lineage, previously isolated from soil. In strains that were close relatives of transconjugants but unable to acquire the plasmid, we observed five defence systems not present in transconjugants that may act as barriers to plasmid acquisition. Our approach demonstrates how auxotrophic donor counter-selection can be deployed at scale to screen PHR in environmental isolates and to investigate the influence of donor identity on plasmid conjugation.

## Data availability

New sequences generated in this study were deposited under the BioProject PRJNA1465300. Accession numbers are displayed in Table S2.

## Introduction

Conjugative plasmids, extrachromosomal genetic elements that encode the genes necessary for their own transfer, are a major driver of horizontal gene transfer in bacterial communities [1], along with other mechanisms, such as transduction and transformation. Conjugation spreads ready-to-use adaptive phenotypes (e.g. xenobiotic metabolism [2], antibiotic resistance [3], toxin resistance [4]) across taxonomic boundaries, enabling rapid adaptation to changing environmental conditions [5]. Conjugation requires physical contact between donor and recipient cells, allowing plasmid transfer via the conjugation pilus [6]. A fundamental limit on plasmid transmission is plasmid host range (PHR), which is predicted to be determined by the combination of microbial host and plasmid genetics [7,8]. PHR is poorly understood for many plasmids [9]. Based upon the taxonomic distribution of hosts, plasmids are often categorized into broad or narrow host range [10]. Broad host range plasmids occur in distantly related taxa [11], even across multiple kingdoms [12,13], while narrow host range plasmids are confined within a species or genus [14]. While PHR is encoded by genetic factors of the plasmid itself [15], it is also affected by the identity of the donor, meaning that the same plasmid in different donors can display differences in the taxonomic identity and range of plasmid recipients [16,17]. Recipients may also affect PHR through mechanisms, including plasmid-mediated entry exclusion [18], defence systems targeting foreign DNA (restriction modification [19], CRISPR-Cas [20]) or altering the cell surface [21,22] that prevent plasmid transfer. Given this complex interplay of donor-plasmid-recipient factors upon PHR, it has proven challenging to predict PHR computationally [23]. Nonetheless, understanding PHR is crucial for clinical surveillance of multidrug resistance plasmids and for selecting plasmids to use in biotechnological applications where tailored delivery of genes is required [24,26]. Accessible, scalable methods for quantifying PHR in non-model bacterial taxa are needed.

Traditional methods for observing plasmid conjugation use donor and recipient strains that have both been tagged with distinct selectable marker genes (e.g. an antibiotic resistance cassette). Transconjugants are then recovered by plating on dual selection medium, selecting for both the recipient’s chromosomal marker gene and the plasmid-encoded trait [27,28]. Such methods are of limited value for studying PHR in natural isolates because they require genetic manipulation of recipient strains, which is often challenging outside of a limited number of model organisms [29]. An alternative approach is to counter-select against the donor strain, bypassing the necessity of genetically modifying recipients. Fluorescently tagging the donor strain and plasmid with distinct fluorophores allows for donors to be separated from transconjugants on the basis of fluorescence using cell sorting [7,30]. This method is powerful because it can be used with unculturable organisms but requires access to expensive state-of-the-art equipment (e.g. FACS). A lower-tech alternative means of counter-selection is to use an auxotrophic donor (i.e. a strain unable to grow in the absence of an essential nutrient). Here, donors can be separated from transconjugants by plating on medium that lacks the relevant nutrient but is supplemented with a selective agent for the plasmid-encoded trait. For example, *Escherichia coli* strains with diaminopimelic acid auxotrophy have been used to transfer cloning plasmids (derivatives of pECE743) into *Bacillus* [31] and the integrative pSET152 plasmid to *Actinomycetes* strains [32]. In addition*,* a d-alanine *Bacillus subtilis* auxotroph was used to transfer an integrative and conjugative element to a range of Gram-positive bacteria [33].

**Fig. 1.**
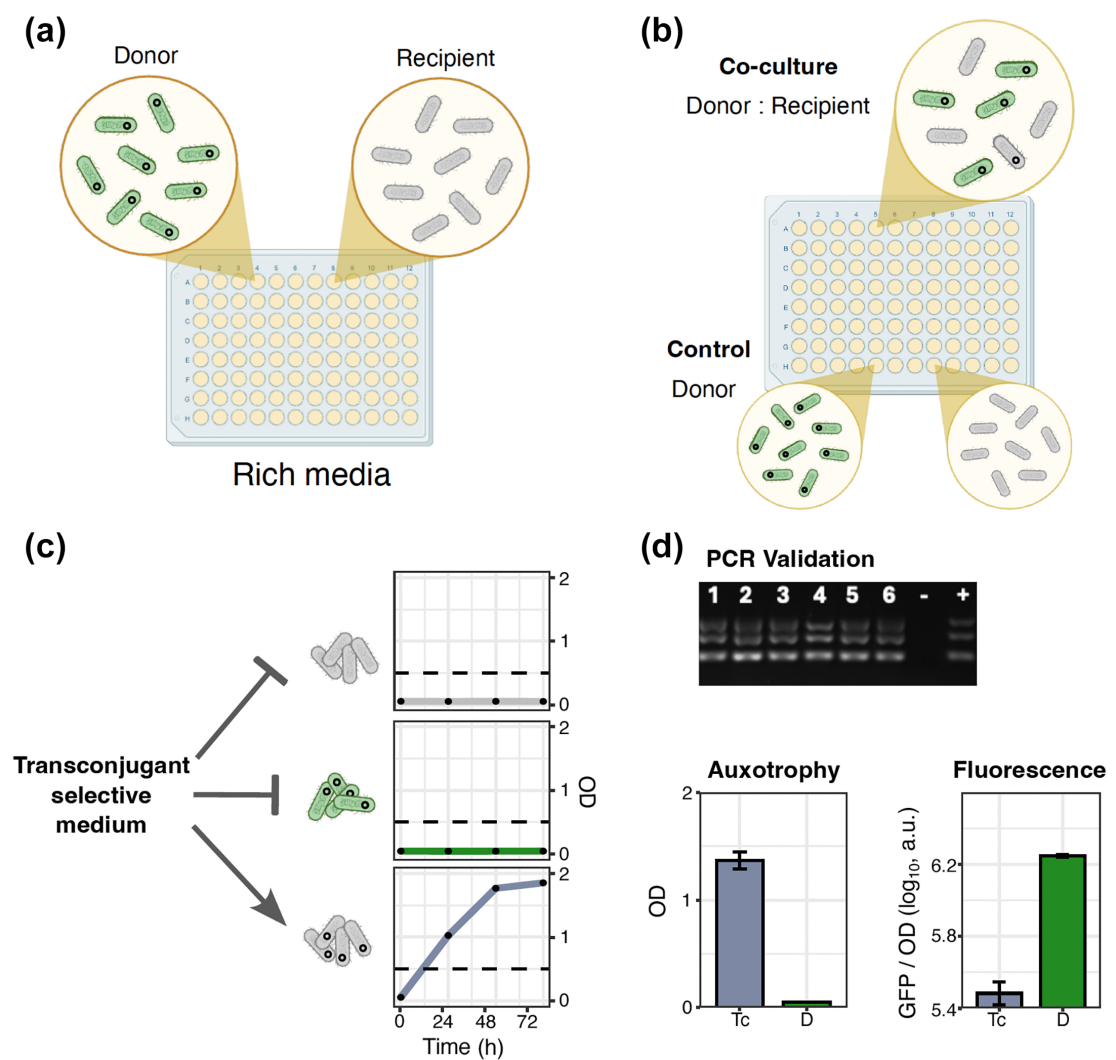
Method workflow. A representative example using *P. putida* KT2440 D*trpD gfp* as the donor of pQ-KAB and *Pseudomonas* isolate D5 as the recipient. (**a**) Donor and recipient are grown independently in rich medium, then (**b**) they are mixed in co-culture in rich medium, maintaining monocultures as controls. (**c**) After 24 h incubation, co-cultures are re-inoculated at three dilutions in Tc-selective medium (M9 medium with 10 mM glucose and 20 mM HgCl_2_), the lack of essential nutrients inhibits donor growth while mercury prevents plasmid-free cells from growing, thus selecting for transconjugants. Wells with optical density (OD_600_) above 0.4 are streaked on plasmid-selective medium (King’s B [44] agar 20 mM HgCl_2_). (**d**) Transconjugants confirmed by plasmid presence by PCR screening, growth on M9 medium with 10 mM glucose (no tryptophan supplementation) and lack of fluorescence. Created with BioRender.com.

In this study, we applied auxotrophic donor counter-selection in a high-throughput screening format to characterize PHR using *Pseudomonas fluorescens* SBW25 D*panB tdTomato* (pantothenate auxotroph) and *Pseudomonas putida* KT2440 D*trpD gfp* (tryptophan auxotroph) donors along with the environmental plasmid pQBR57-KAB (Fig. 1). pQBR57 is a conjugative megaplasmid (307 kb) isolated from the sugar beet phytosphere [34] that naturally contains a mercury resistance operon within the Tn5042 transposon [28]. The transfer, maintenance and fitness cost properties of pQBR57 within *P. fluorescens* have been thoroughly characterized [35,38]. pQBR57-tphKAB (hereafter pQ-KAB) is an engineered version of pQBR57 equipped with transport and catabolic genes for the assimilation of terephthalic acid (tphKAB) [39]. Terephthalic acid is the aromatic monomer of polyethylene terephthalate plastic. pQ-KAB has shown promise as a potential vector for genetic bioaugmentation-mediated bioremediation of soils [40]. We assessed pQ-KAB’s PHR from each donor into a panel of recipients comprising 101 diverse bacterial isolates isolated from potting soil. We have shown that our method can be used to rapidly screen for plasmid transfer to unlabelled culturable environmental soil bacteria. Moreover, we have shown that PHR varies between donor strains and identified variation in defence systems among potential recipients that may limit plasmid acquisition.

## Methods

### Donor strain construction

*P. fluorescens DpanB* SBW25 is a pantothenic acid (vitamin B5) auxotroph obtained from Rainey *et al*. (1998) [41]. *P. putida DtrpD* was constructed by using the pK18mobsacB [42] vector, which incorporated a gentamicin cassette at the locus of *trpD* via homologous recombination and selection of double crossover with sucrose counter-selection. *P. putida DtrpD* was fluorescently tagged with a constitutively expressed GFP cassette using the same delivery vector and integrating at the *fpvA* locus. *P. fluorescens* was tagged with a tdTomato-expressing cassette using the mini-Tn7 system [43] to insert at the chromosomal attTn7 site. The plasmid pQBR57-tphKAB (pQ-KAB) is from Marquiegui-Alvaro *et al*. (2025) [40] and was horizontally transferred to the auxotroph via conjugation from a non-fluorescent *P. putida* donor. Briefly, plasmid-carrier and auxotroph were grown in coculture in King’s B [44] (KB) medium, composed of 20 g l^−1^ Bacto proteose peptone No.3 (Difco), 1.5 g l^−1^ potassium phosphate dibasic trihydrate (Merck), 1.5 g l^−1^ magnesium sulphate heptahydrate (Merck) and 10 g/l glycerol (Thermo Fisher Scientific), for 24 h at 30 °C. Then, cocultures were plated on plasmid-selective solid medium composed of 1X KB, agar (Thermo Fisher Scientific) and HgCl_2_ 20 mM (Merck). Colonies with fluorescence were picked and their auxotrophy confirmed by their lack of growth in M9 medium with 10 mM glucose, d-(+)-glucose (Merck).

To characterize their auxotrophy, they were grown in M9 medium with 10 mM glucose and a gradient of essential nutrients. l-Tryptophan (Merck) was dissolved at 10 mg ml^−1^ in 1M HCl and d-pantothenic acid hemicalcium salt (Merck) was dissolved at 50 mg ml^−1^ in Milli-Q water. These solutions were diluted in M9 medium with 10 mM glucose across a range of concentrations and three colonies of each donor, which had previously grown in KB for 18 h at 30 °C and been washed twice with M9 medium to remove traces of essential nutrients, were diluted 1/100 and grown for 24 h at 30 °C.

### Microbial soil isolates, isolation and characterization

One hundred and one isolates were obtained directly from compost potting soil. We added 10 g John Innes No. 2 compost soil in 30 ml universal glass vials and added 10 ml of M9 salt solution (Merck) and 20 glass beads. After vortexing for 1 min, the soil wash was plated onto KB agar plates. These plates were then incubated at 30 °C with 80% humidity for 48 h. Subsequently, we selected individual bacterial colonies that we grew in KB medium and stored in 25% glycerol (Thermo Fisher Scientific) at −80 °C. We systematically filtered out the bacterial isolates with native resistance to mercury by growing them for 48 h at 30 °C in 96-well microplates (Corning) with KB and 20 mM HgCl_2_.

We additionally characterized the bacterial isolates through 16S rRNA amplicon sequencing. We performed PCR using Phusion High-Fidelity polymerase (Thermo Fisher Scientific) and a specific set of primers designed to target the 16S rRNA gene (27F and 1492R in Table S1, available with the online version of this article). The PCR protocol included the thermocycling programme: initial denaturation at 95 °C for 2 min, followed by 35 cycles of denaturation at 95 °C for 30 s, annealing at 55 °C for 30 s, extension at 72 °C for 1 min and 40 s and a final extension at 72 °C for 5 min. The resulting 16S rRNA gene amplicon was sequenced by GENEWIZ, utilizing both the forward and reverse primers to ensure adequate coverage of the 16S rRNA gene. The sequenced contigs were aligned using the R package ‘sangeranalyseR’ and classified through blastn analysis [45]. Multiple alignments of amplicon sequences were performed using muscle in mega 11 with the following parameters: Cluster Method- Iterations 1, 2: UPGMA; Cluster Method- Other Iterations: UPGMA; Min Diag Length- Lambda: 24 [46]. The phylogenetic analysis was conducted using IQ-TREE with maximum likelihood, automatic thread assignment, 1,000 ultrafast bootstrap replicates, 1,000 aLRT (approximate likelihood-ratio test) replicates and a specific branch-and-bound search strategy, considering non-parametric rate heterogeneity [47]. To visualize the phylogenetic trees, we used the R packages ‘phylotools’ and ‘ggtree’ [48,49].

### Plasmid host range screening

Three biological replicates of each donor (*P. fluorescens DpanB tdTomato* pQ-KAB and *P. putida DtrpD gfp* pQ-KAB) were picked by streaking in KB agar with 20 mM HgCl_2_. These were grown in 5 ml of KB with 20 mM HgCl_2_ at 30 °C for 18 h at 180 r.p.m. The cultures were washed with KB to remove any traces of HgCl_2_. For the soil isolates, they were grown in KB in 96-well round plates at 30 °C for 18 h. Donors and recipients were inoculated at a 1 : 5 c.f.u. ratio in favour of recipients in KB and grown at 30 °C for 18 h (Fig. S1). Then, the plates were washed with M9 minimal medium to remove any traces of essential nutrients (pantothenic acid and tryptophan) and inoculated in Tc-selective medium (M9 medium with 10 mM glucose and 20 mM HgCl_2_) at four dilutions (neat, 1/10, 1/100 and 1/1,000). These cultures were grown at 30 °C for 72 h. The co-cultures that significantly grew, compared to the single-strain controls (i.e. OD_600_ >0.4) were streaked in KB agar ±20 mM HgCl_2_ and single colonies were screened for plasmid carriage via multiplex PCR using Taq Green Hot Start (Thermo Fisher Scientific) and targeting the *tphK (142 bp*), *uvrD (510 bp)* and *merA (795 bp*) genes of the plasmid. The PCR protocol consisted of 5 min at 95 °C, followed by 30 cycles of 30 s at 95 °C, 30 s at 58 °C and 1 min at 72 °C, followed by final extension for 5 min at 72 °C. In addition, these putative transconjugant colonies were grown in M9 medium with 10 mM glucose and in KB to confirm lack of auxotrophy and fluorescence, respectively. Therefore, to assign the transconjugant label, three requirements had to be met: (i) PCR product with three targeted genes, (ii) OD_600_ >0.5 after 24 h incubation in M9 10 mM glucose and (iii) fluorescence below 10^6.4^ (arbitrary units, a.u.) for green and 10^4.5^ (a.u.) for red in KB rich media. Screening data accessible in File S1.

### Genome sequencing, phylogenomic and defence-systems analysis of transconjugants

After confirmation of transconjugants via PCR, auxotrophy and fluorescence assays, single colonies of transconjugants were grown in KB medium with selection (20 mM HgCl_2_) or without selection at 30 °C for 16 h and 180 r.p.m. In addition, closely related neighbours that did not acquire the plasmid were included and grown without selection. Cell count was estimated via c.f.u. and 5×10^9^ cells were washed with M9 medium and resuspended in Zymo DNA/RNA Shield (Zymo Research). These cells were taken preferentially from KB with selection, but when growth was poor with selection, cultures grown without selection were used. Sequencing was outsourced to Plasmidsaurus Ltd. Briefly, they created an amplification-free long-read sequencing library and sequenced with Oxford Nanopore. In-house assembly involved the removal of 5% worst reads with Filtlong v0.2.1 (default parameters) and was assembled with Flye v2.9.1 with parameters selected for high-quality ONT reads. The assemblies were polished with Medaka v1.8.0, annotated with Bakta v1.11, contig analysis with Bandage v.0.8.1 and genome completeness and contamination check with CheckM v 1.2.2.

From the resulting assemblies (Table S2), chromosomal contigs were submitted to the TYGS web server (Type Strain Genome Server)^50^ for taxonomy assignment and phylogenomic inference. TYGS infers phylogenomic trees using the Genome blast Distance Phylogeny (GBDP) method and automatically includes reference type strains from its database for taxonomic assignment. Pseudo-bootstrap support values were calculated from 100 replicates. *P. fluorescens* and *P. putida* were explicitly included as additional reference strains in the analysis. To determine the number and identity of defence systems in these isolates, DefenseFinder v2.0.0 was used with default parameters on the Bakta-annotated genomes (from the sequencing provider) of each isolate.

## Results

### Engineering and validation of auxotrophic donor cells

**Fig. 2.**
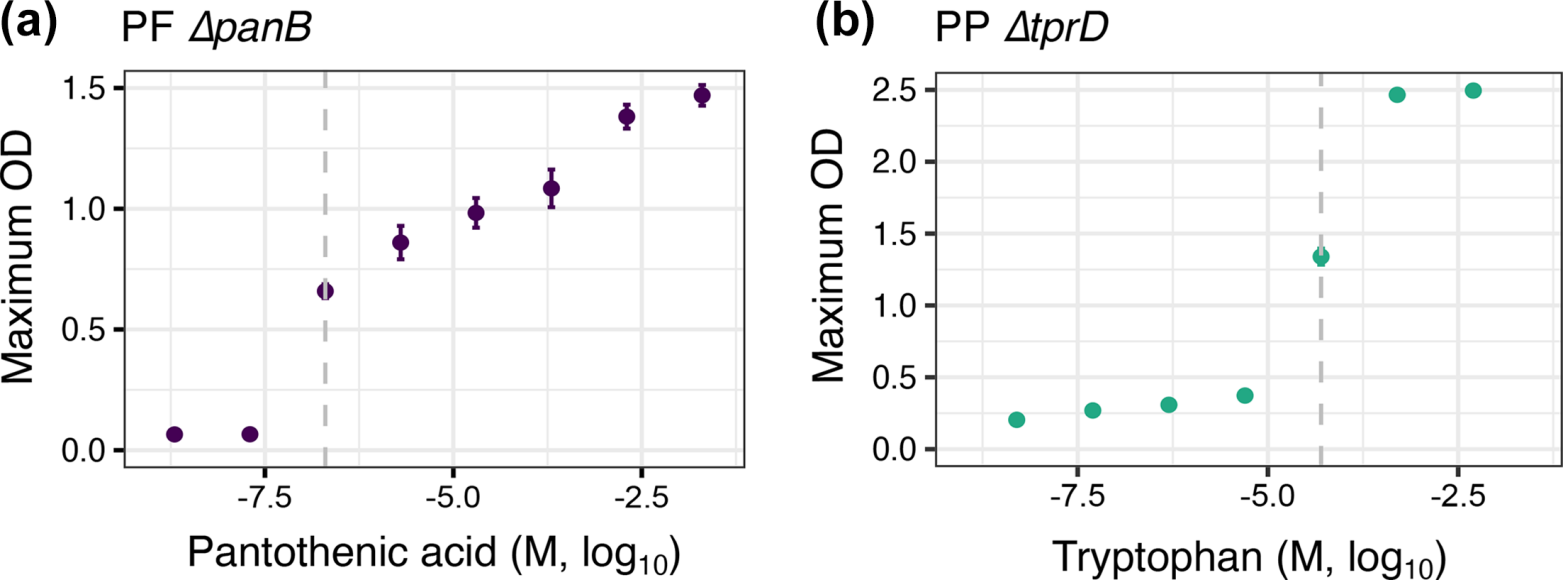
Auxotroph confirmation. (**a**) Pantothenic acid auxotrophy of *P. fluorescens DpanB* (PF D*panB*) in a gradient of pantothenic acid concentrations, (**b**) Tryptophan auxotrophy of *P. putida DtrpD* (PP D*trpD*) in a gradient of tryptophan concentrations; both in minimal medium supplemented with 10 mM glucose for 24 h at 30 °C.

We first characterized the auxotrophic strains we used as donors. The *P. fluorescens* pantothenate auxotroph, *P. fluorescens DpanB* (hereafter PF D*panB*), was originally constructed by homologous recombination [41]. Production of pantothenate (vitamin B5) begins with PanB, a ketopantoate hydroxymethyltransferase which catalyzes the conversion of α-ketoisovalerate to α-ketopantoate. We validated the pantothenate auxotrophy by quantifying growth in liquid minimal medium supplemented with a range of pantothenate concentrations (Fig. 2a), confirming that PF D*panB* is unable to grow at concentrations below 20 nM. We constructed the *P. putida* tryptophan auxotroph, *P. putida DtrpD* (hereafter PP D*trpD*), by homologous recombination with a suicide vector [42]. Tryptophan is synthesized from chorismate via the action of *trpDC* operon [51], where TrpD encodes an anthranilate phosphoribosyltransferase which catalyzes the essential step in tryptophan biosynthesis [52]. We validated the auxotrophy by quantifying growth in minimal medium supplemented with a range of tryptophan concentrations (Fig. 2b), confirming that PP D*trpD* is unable to grow at concentrations below 1 mM. Both auxotrophic donors, PF D*panB* and PP D*trpD*, were then chromosomally tagged with tdTomato or GFP, respectively, under a constitutive promoter. pQ-KAB was then conjugated to each donor from *P. fluorescens* SBW25.

### Taxonomic diversity of soil isolates used as potential recipient panel

**Fig. 3.**
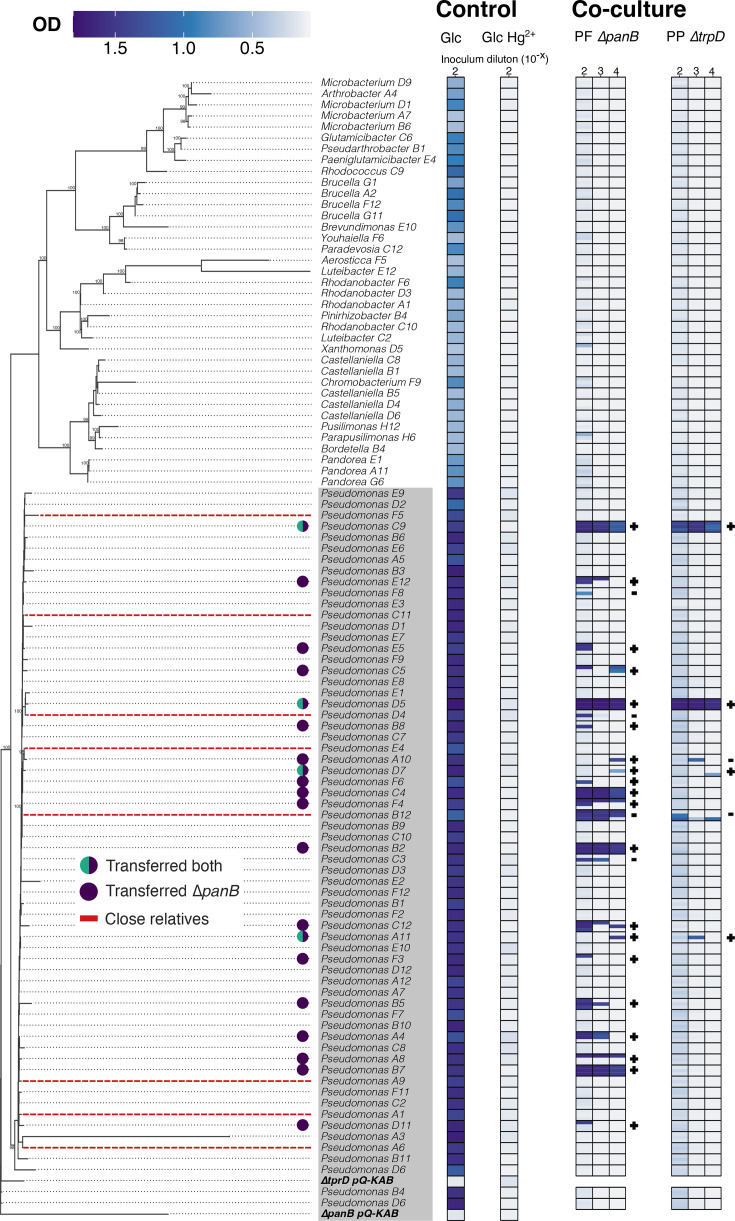
Phylogenetic tree of isolates and growth in Tc-selective medium. Phylogenetic tree of soil isolates based on full 16S rRNA sequence, branch length has been scaled down for non-*Pseudomonas* and PF D*panB* to help visualization. The grey area represents *Pseudomonas* genus. Purple and bicolour circles show the detected transconjugants from PF D*panB* or both donors, respectively, close relatives that did not acquire the plasmid are marked with a red dotted line. The heatmaps adjacent to the tree represent the maximum OD_600_ in a 72 h incubation. The first heat map column is from monocultures of each isolate and donor in M9 10 mM glucose (positive control), followed by monocultures in Tc-selective medium M9 10 mM 20 mM HgCl_2_ (negative control). Then, the three columns under PF D*panB* represent the co-culture of isolate and corresponding donor, at three inoculation dilutions in Tc-selective medium, while the remaining three are the co-culture of each isolate and PP D*trpD* at three inoculation dilutions. For the co-culture heatmaps, three replicates are shown for each isolate-donor combination. Next to the putative transconjugant growth, the outcome of the post-hoc tests (plasmid-specific PCR, validate auxotrophy and lack of fluorescens, Fig. S3 and File S1), where ‘+’ indicates a validated transconjugant, while ‘-’ implies at least one post-hoc test failed. Node labels show UFBoot bootstrap support values (IQ-TREE ultrafast bootstrap); only nodes meeting the recommended support thresholds of SH-aLRT≥80% and UFBoot≥95% are labelled.

We isolated 128 pure colonies from KB agar plates inoculated with potting soil wash. Prior work suggests that this method captures a diversity of culturable bacterial strains [53]. The isolates were then screened for their ability to grow in minimal medium with glucose and sensitivity to Hg^2+^ to filter out isolates unable to grow in the culture conditions of our screen or with native mercury resistance. These requirements ensured that only plasmid-carrying recipients could grow in Tc-selective medium (M9 medium with 10 mM glucose and 20 mM HgCl_2_) due to recipients’ sensitivity to mercury and the donors’ auxotrophies (Fig. 3). These requirements resulted in 101 isolates that were taxonomically characterized by sequencing the full 16S rRNA gene (Fig. 3). 64 isolates belonged to *Pseudomonas,* while 37 belonged to a diverse range of bacterial taxa, including *Bacillus*, *Pandorea*, *Rhodanobacter*, *Xanthomonas* or *Brucella*.

### pQ-KAB preferentially conjugates into *Pseudomonas* recipients

**Fig. 4.**
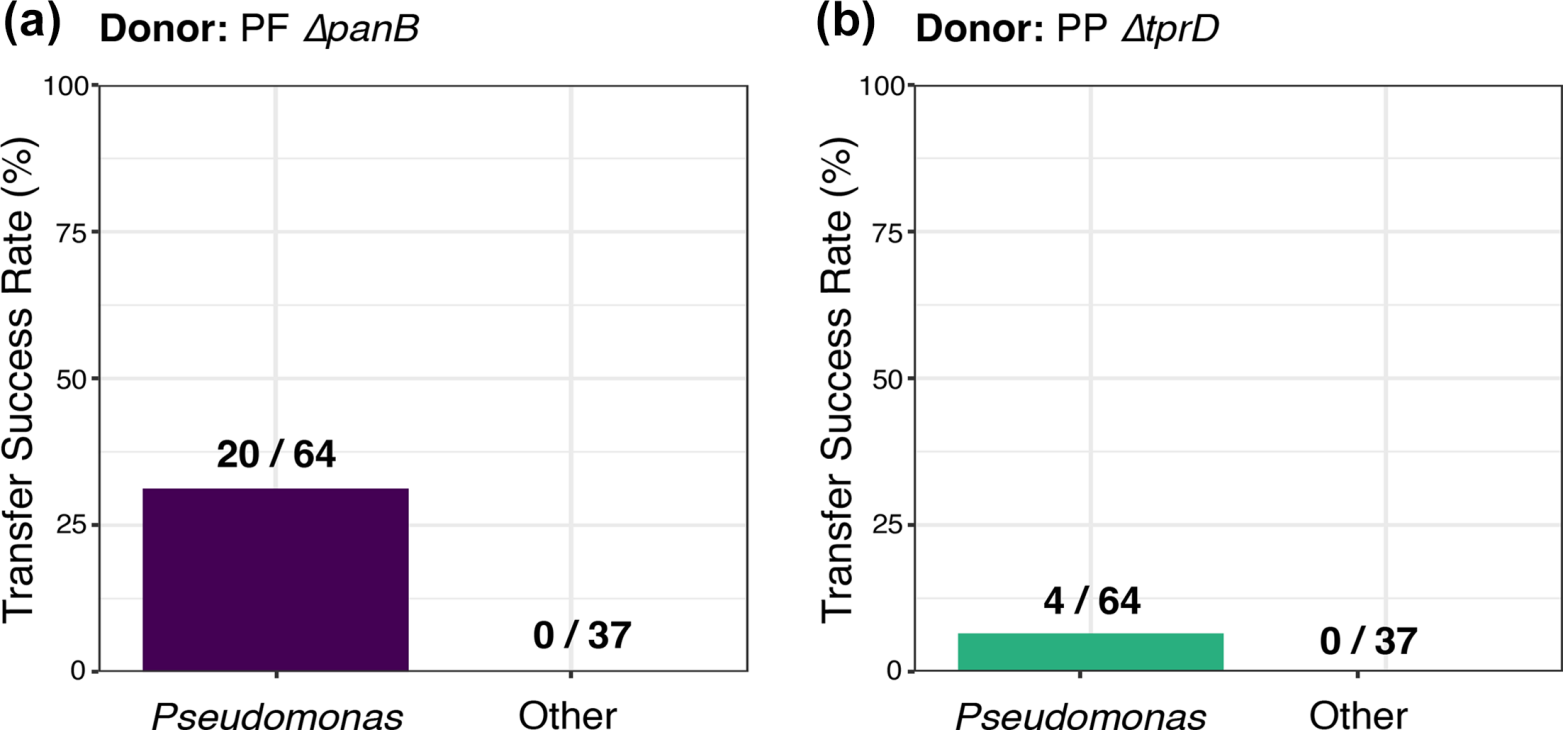
Transfer success rate is donor-dependent. (**a**) Transfer success rate within-genus (*Pseudomonas*) and outside-genus (non-*Pseudomonas*) with PF D*panB* pQ-KAB as the donor and (**b**) with PP D*trpD* pQ-KAB.

Our PHR screen identified six putative transconjugants from PP D*trpD* and 24 from PF D*panB* (i.e. growth exceeded threshold OD_600_ >0.4) (Fig. 3, co-culture heat maps). All of the transconjugants belonged to *Pseudomonas*. Post-hoc analyses (PCR, lack of auxotrophy and fluorescence) confirmed four transconjugants from PP D*trpD* and 20 transconjugants from PF D*panB* (shown in Fig. 3 with a ‘+’ sign). Thus, PF D*panB* transferred the plasmid to 31.6% of the *Pseudomonas* isolates (20/64) and PP D*trpD* to 6.3% (4/64) (Fig. 4a and b). The PHR of pQ-KAB from PP D*trpD* was a subset of the PHR observed from PF D*panB* (*Pseudomonas*-A11, *Pseudomonas*-C9, *Pseudomonas*-D5 and *Pseudomonas*-D7, marked with a bicolour circle in Fig. 3). Plasmid transfer success did not vary with phylogenetic distance of the recipient from the donor (logistic regression, *P*-value=0.17 for PF D*panB* and *P*-value=0.26 for PP D*trpD*).

### Transconjugants lack specific defence systems compared to close relatives

To identify transconjugants at the species level, we obtained whole genome sequences for each of the 20 PCR-confirmed transconjugants. We also obtained whole genome sequences for eight isolates that were phylogenetically closely related to transconjugants (according to their 16S rRNA sequence) but were unable to acquire pQ-KAB from either donor in our PHR screen (labelled as close relatives in Fig. 3). Genome assembly resulted in a single chromosomal contig for those close relatives that did not acquire the plasmid, while for transconjugants most had a secondary 313 kb-long contig for pQ-KAB. Some transconjugants had additional contigs: *Pseudomonas*-B2 (161 kb) and *Pseudomonas-*C4 (51 kb), possibly indicating the presence of other plasmids, while for 3 of the confirmed transconjugants (*Pseudomonas*-A10, -A11 and -D7), the pQ-KAB contig was not detected. This may be explained by plasmid loss during the non-selective growth conditions used to obtain sufficient biomass for sequencing. Transconjugant cultures were grown both with and without selection; however, when growth under selection was impaired or insufficient, cultures without selection were submitted for sequencing.

**Fig. 5.**
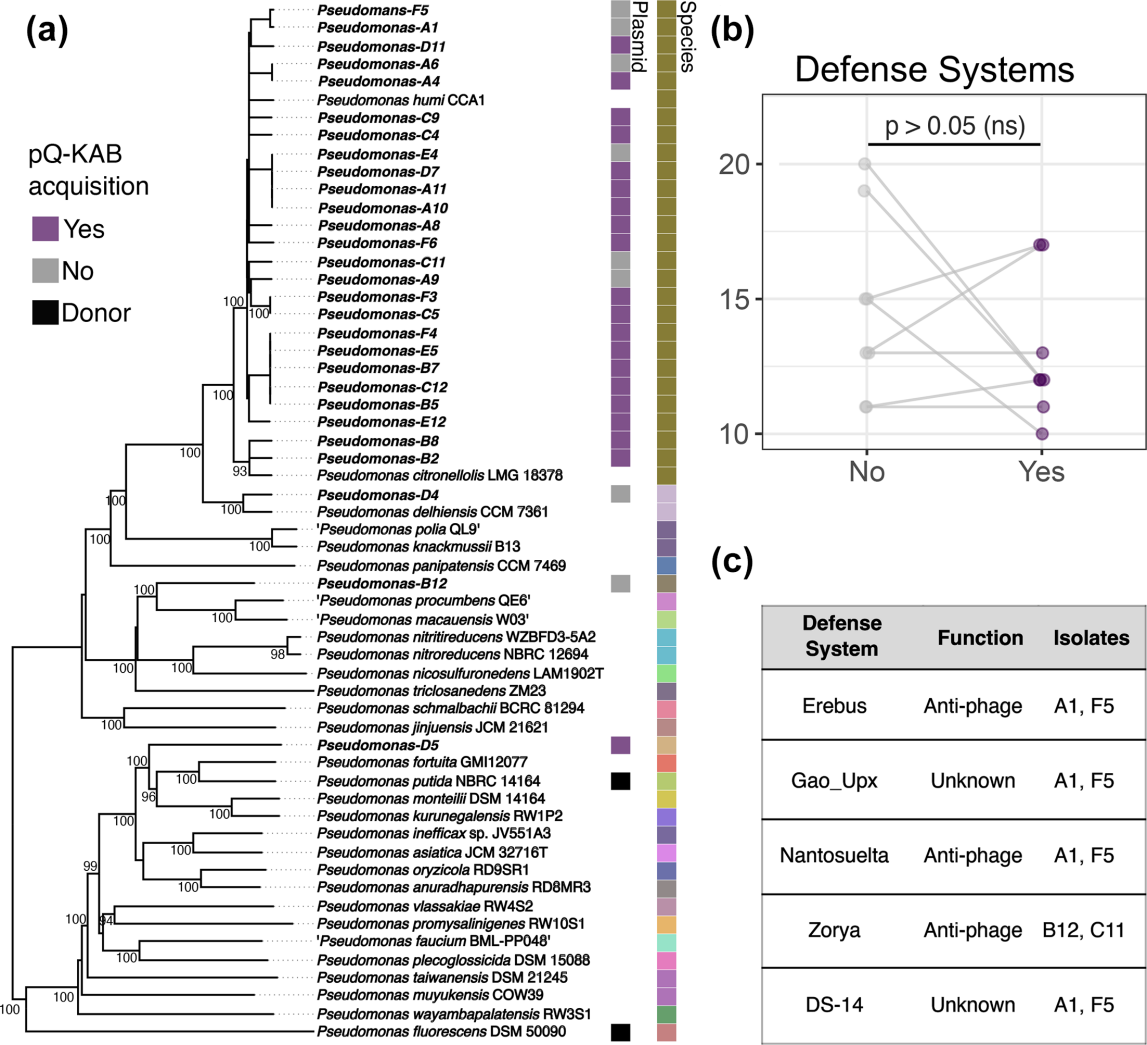
Taxonomic identification of transconjugants and closely related neighbours and their defence systems. (**a**) Phylogenomic tree of pQ-KAB transconjugants and closely related isolates that did not acquire the plasmid. Tree was inferred using the GBDP method on the TYGS web server[50], with chromosomal contigs as input. Reference type strains were automatically included by TYGS for taxonomic assignment. Transconjugants and non-transconjugant close relatives are indicated by pink and grey squares and donors with black squares. Adjacent to the plasmid carriage column, species grouping is displayed with an individual colour for each group. Assignment is displayed. Branch lengths reflect GBDP distances; numbers at nodes indicate pseudo-bootstrap support values based on 100 replicates [70]. (**b**) Total number of defence systems identified with DefenseFinder [71,72] between the isolates that could acquire the plasmid and their close relatives, significance from paired sample t-test. (**c**) Defence systems identified exclusively in the isolates that could not acquire the plasmid and their putative function.

Most transconjugants clustered with *Pseudomonas citronellolis* LMG 18378 and *Pseudomonas humi* CCA1 (Fig. 5A). *P. citronellolis* is a bacterium found in soil and plant surfaces [54], and *P. humi*, originally isolated from leaf soil in Japan [55], has since been proposed as a later heterotypic synonym of *P. citronellolis* based on whole-genome comparisons [56]. Interestingly, *Pseudomonas-*D4 clustered next to *Pseudomonas delhiensis* CCM 7361, which was isolated from a fly ash dumping site in India [50], while isolates *Pseudomonas*-B12 and D5 do not clearly cluster with any *Pseudomonas* strain.

We compared the defence system content of transconjugants with close relatives that could not acquire the plasmid. Although the groups did not differ in the number of defence systems (paired sample t-test, *P*-value=0.33) (Fig. 5B), five defence systems were found exclusively in the close relatives that could not acquire the plasmid (Fig. 5C). Specifically, these were the characterized anti-phage defence systems Erebus, Nantosuelta and Zorya, as well as two defence systems with unknown mechanisms (Gao_Upx and DS-15).

## Discussion

Here, we report the application of an auxotrophic donor counter-selection strategy in a scalable PHR screening format, using two auxotrophic donors to screen 101 soil-isolated environmental bacteria for their ability to acquire pQ-KAB by conjugation. We identified 20 strains of *Pseudomonas* capable of acquiring pQ-KAB without the need to genomically tag any of the recipient strains. Our data show that pQ-KAB’s PHR is likely limited to within the *Pseudomonas* genus. In addition, pQ-KAB’s PHR was affected by the donor identity (Fig. 4A and B) and the defence systems encoded by potential recipients, which may limit pQ-KAB transfer.

Our findings corroborate previous experiments using wild-type pQBR57 showing that these plasmids preferentially conjugate into *Pseudomonas* species [36,57]. In contrast, a study by Hall *et al.* (2020) [35] using *P. fluorescens* as the donor of pQBR57 reported, via epicPCR targeting the *merA* gene, the transfer of the mercury-resistance cassette beyond *Pseudomonas*, including Burkholderiales, Rhizobiales and even Bacillales. This broader host range could be due to the target gene, *merA*, being located on a transposon (Tn5042). This could have enabled *merA* to relocate to the chromosome during transient plasmid acquisition by these species or onto other mobile genetic elements that these taxonomic orders can then acquire [57,58]. In addition, our findings emphasize the previously reported role of donor identity upon PHR [17]. *P. putida* has a lower stability and conjugation rate of pQBR57 when compared to *P. fluorescens*,^59^ which could explain the difference in their ability to transfer pQ-KAB (Fig. 4). Our data suggest that *P. fluorescens* is a more efficient plasmid donor, consistent with its role as a plasmid reservoir in co-culture with *P. putida* in potting soil [37].

In general, the likelihood of conjugation is expected to decline with increasing phylogenetic distance between donor and recipient [60]. Although we observed that pQ-KAB appears restricted to *Pseudomonas*, we observed no significant phylogenetic signal in PHR. Such patterns are likely to be more apparent at higher levels of taxonomic classification (e.g. class, orders) [60]. At lower taxonomic levels (e.g. genus), phylogenetic distance shows a less strong correlation with the likelihood of transfer [61]. Within the genus, the presence of defence systems [61] or other plasmids [18] appears to be a more important determinant for the success of plasmid transfer. Within *Pseudomonas*, most of the pQ-KAB transconjugants clustered within *P. citronellolis* lineage (Fig. 5A), suggesting that pQ-KAB is a relatively narrow host range plasmid. Moreover, the findings suggest that the natural host of the parental pQBR57, which is unknown due to its isolation by exogenous capture from a sugar beet field [34], may have been a strain of *P. citronellolis*.

Comparison of defence systems in closely related strains that did or did not receive pQ-KAB supported a potential role for bacterial immunity in PHR here. Although the total number of defence systems did not differ between transconjugants and non-transconjugants (Fig. 5B), we identified five systems that were exclusively found in close relatives unable to acquire pQ-KAB (Fig. 5C). This dissociation could indicate targeting of pQ-KAB by these defence systems, though given the small sample size, further work would be needed to confirm this. Where known, the mechanism of action of these defence systems (e.g. Zorya) is to digest foreign nucleic acid via nuclease effectors [62,64]. Although some defence systems are thought to exclusively target phage DNA and have no effect on conjugation (e.g. Zorya) [64], there are other systems where the specificity of targeted Mobile Genetic Elements (MGEs) remains unclear. That defence systems impose selection on conjugative plasmids is clear from plasmids encoding anti-defence systems in their leading region [65]. Functional validation would be needed to determine whether these systems directly impede pQ-KAB acquisition, for example, by targeting the incoming leading strand during conjugation.

Our PHR screening method has several advantages relative to existing conjugation protocols. First, it bypasses the need to genetically manipulate the recipient, enabling testing of PHR in environmental bacteria. As such, this protocol could be used to identify plasmid-donor pairs capable of transferring genetic material to non-model organisms for investigating their biotechnological potential [66,68]. Second, our method is relatively high throughput, since it can be carried out in 96-well plates or 364-well plates, with the complete workflow taking only 5 days to perform (Fig. 1). Third, it is cost-effective and reliant on common lab consumables and equipment (OD plate reader, incubator, thermocycler). The more expensive techniques used (e.g. whole-genome sequencing) are not essential, although they do provide an additional layer of characterization of transconjugants. The main limitation of our method is that recipient strains must fulfil certain criteria: (i) lab cultivability; (ii) sensitivity to the plasmid selective agent; (iii) prototrophic growth; (iv) competitive growth alongside the donor strain in co-culture conditions. Some recipient strains may grow poorly or be outcompeted in co-culture, reducing the opportunity for conjugation events to occur and limiting transconjugant recovery independently of any genuine plasmid incompatibility. In addition, we observed a low rate of false positives, 20% (Fig. 2, total 6/30; 4/24 from *P. fluorescens* and 2/6 from *P. putida*), whereby non-plasmid carrying recipients, or donors, grew in Tc-selective medium. We hypothesize that growth in these co-cultures may be the result of sufficient detoxification by donors by reducing mercury, followed by growth of plasmid-free recipients. Alternatively, disruption of cell membrane integrity by mercuric ions [69] could release sufficient pantothenate or tryptophan to allow donor growth. However, the additional PCR, auxotrophy and fluorescence screenings can rapidly filter out such false positives.

Overall, our PHR screening method provides a robust and accessible platform for assessing the effect of donor strains on PHR across panels of non-model environmental isolates without requiring genetic manipulation of potential recipients. By enabling enrichment and isolation of transconjugants, it facilitates downstream genomic and phenotypic characterization of newly formed plasmid–host associations. As such, this approach offers a valuable tool for dissecting the ecological and evolutionary constraints shaping plasmid spread and for identifying donor–recipient and plasmid-recipient combinations with potential utility in environmental microbial biotechnology.

## Supplementary material

10.1099/mic.0.001737 Supplementary Material 1.

10.1099/mic.0.001737 Supplementary Material 2.

## Abbreviations

ALRT: approximate likelihood-ratio test
a.u.: arbitrary unit
GBDP: Genome BLAST Distance Phylogeny
KB: King B
MGE: Mobile Genetic Element
OD: optical density
PHR: plasmid host range
Tc: transconjugant
