# Supplementary material for: Using auxotrophic donor strains to explore pQBR57 plasmid host range among environmental soil bacterial isolates
Source: Microbiology (Reading). 2026 Jul 10;172(7):001737. doi: 10.1099/mic.0.001737 (PMC13367071; doi:10.1099/mic.0.001737)
Supplement: Supplementary Material 2. [file mic-172-01737-s002.pdf]

Supplementary Figures

Supplementary Figure 1. Growth of isolates in mono-culture and co-culture with each donor in KB medium. OD_{600} reading after 24-hour incubation at 30°C. (**A**) Isolates in co-culture with Δ*panB* in triplicates (purple circles) with the average shown with a horizontal purple line, the dotted line represents the OD_{600} of Δ*panB* in mono-culture while the triangles are the OD_{600} of each isolate in mono-culture. (**B**) Analogous to (A) but with Δ*trpD* as donor.

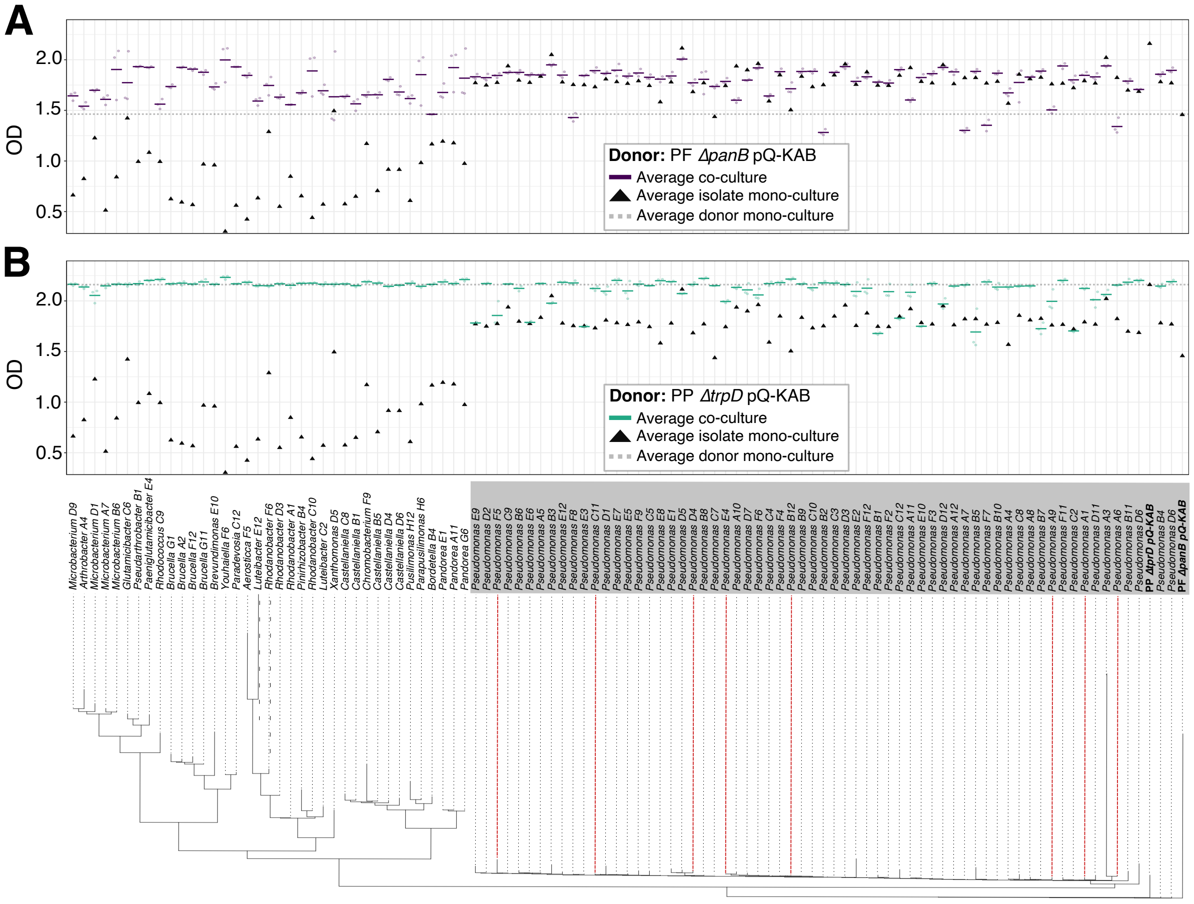

Supplementary Figure 2. PCR control of plasmid-specific primers for all isolates. Each isolate was screened for plasmid carriage and for a chromosomal target in SBW25, specifically targeting 16S rRNA in a fragment suitable for restriction fragment length polymorphisms to differ *P. fluorescens* from other group I *Pseudomonas*[73]. Most *Pseudomonas* isolates have a PCR product for the SBW25 chromosomal target. Importantly, none of the isolates give the characteristic three-band pattern of pQ-KAB carriage. Some non-*Pseudomonas* isolates produce a PCR product with the primers targeting pQ-KAB (B12 and F2), however, the size is far greater than pQ-KAB-derived bands. For naming convention in *Pseudomonas*, each isolate's well is used (i.e. *Pseudomonas*-B2 is shown as B2); while for non-Pseudomonas the conversion of well to identity can be found in **Supp Table 3**. PCR conditions can be found in Materials and Methods, and primer sequences in **Supp Table 1**.

Pseudomonas isolates

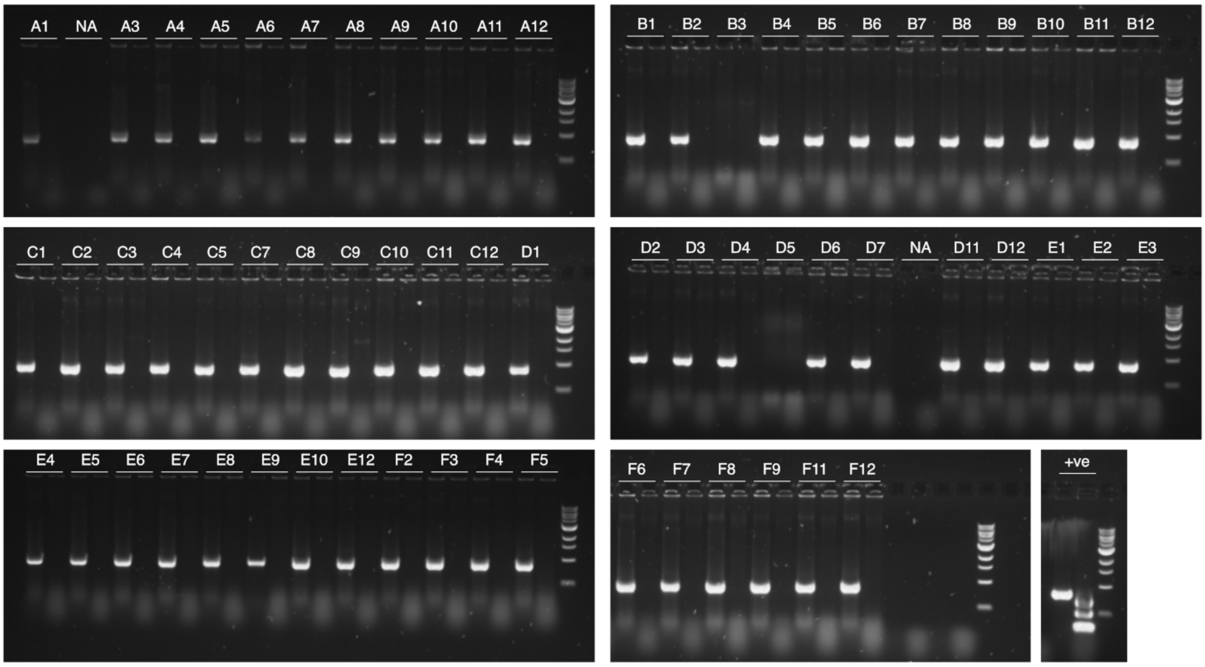

Non-*Pseudomonas* isolates

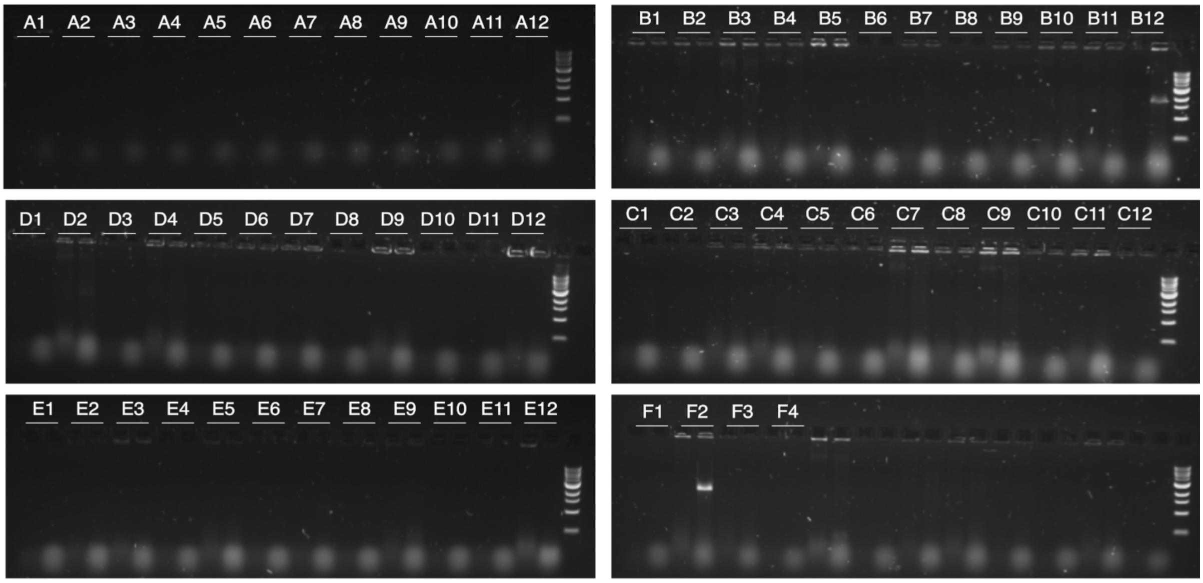

Supplementary Figure 3. PCR screen of *Pseudomonas* putative transconjugants. Each putative transconjugant well was streaked in KB and KB + 20 μM $HgCl_2$ (labelled Hg20). Where possible, two colonies from each medium were screened with plasmid-specific multiplex PCR. For naming convention, each isolate's well is used (i.e. *Pseudomonas*-B2 is shown as B2). Donors are labelled as "p" (SBW25) and "d" (KT2440), followed by their replicate number (n = 3). Controls with donor monocultures are included. PCR conditions can be found in Materials and Methods, and primer sequences in **Supp Table 1**.

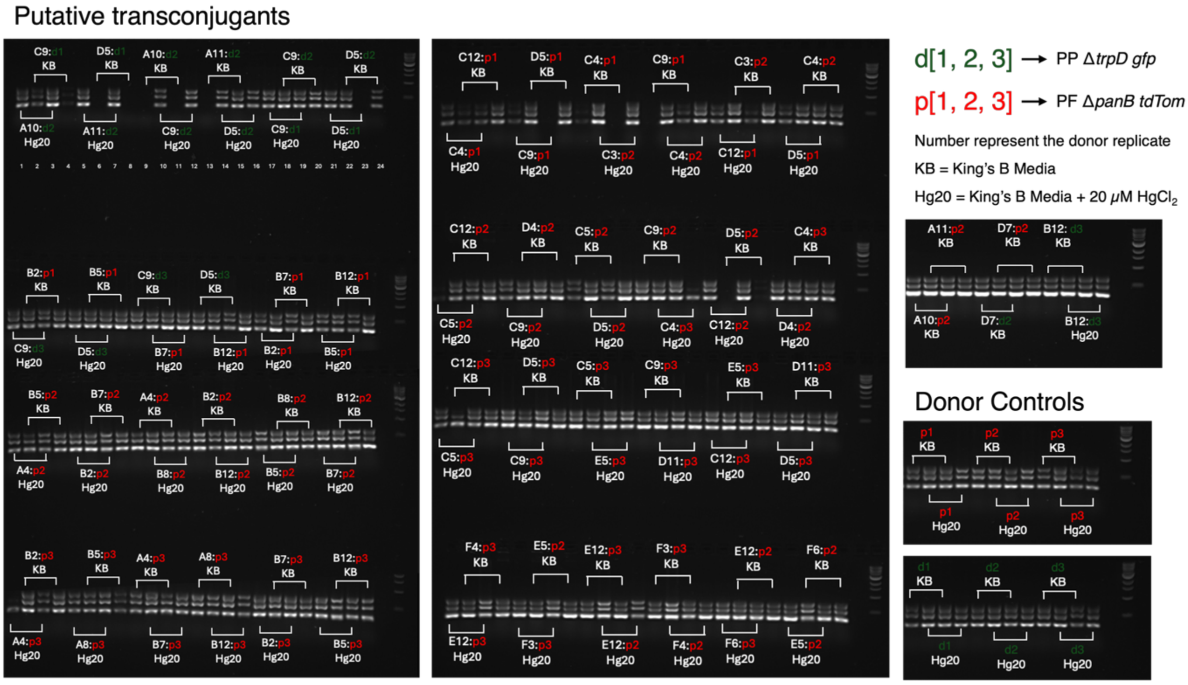

Supplementary Table 1. Primers sequence.

Target	Direction	Sequence (5' - 3')
pQBR57_tphK	fw	ATGCTCCCGGAGTCCAAG
pQBR57_tphK	rev	CCATATAGCTAATTGCTTGGGTATC
Tn5042_merA	fw	TGCAAGACACCCCCTATTGGAC
Tn5042_merA	rev	TTCGGCGACCAGCTTGATGAAC
pQBR57_uvrD	fw	CTTCGAAGCACACCTGATG
pQBR57_uvrD	rev	TGAAGGTATTGGCTGAAAGG
ISPpu10	fw	ATGGCAATGTCCGCAATCC
ISPpu10	rev	CGGAAGCCTCTGAACACG
27F	fw	AGAGTTTGATCMTGGCTCAG
1492R	rev	CGGTTACCTTGTTACGACTT
SBW25	fw	ACTGCATTCAAAACTGACTGA
SBW25	rev	AATCACACCGTGGTAACCG

Supplementary Table 2. Output of whole-genome assemblies from transconjugants and close relatives, along with their BioSample identification for their chromosome available at NCBI. The 313-kb contig represents pQ-KAB in all the samples, while coverage values were obtained from the sequencing provider.

Label	Length	Replicon	Coverage	BioSample
Pseudomonas-A1	7,146,190	Chr	28.9x	SAMN59779553
Pseudomonas-A6	7,027,882	Chr	89.8x	SAMN59779554
Pseudomonas-A9	6,881,729	Chr	45.9x	SAMN59779555
Pseudomonas-B12	5,965,450	Chr	47.7x	SAMN59779556
Pseudomonas-C11	7,150,214	Chr	75.6x	SAMN59779557
Pseudomonas-D4	7,290,528	Chr	61.4x	SAMN59779558
Pseudomonas-E4	6,741,218	Chr	99.9x	SAMN59779559
Pseudomonas-F5	6,947,941	Chr	84.4x	SAMN59779560
Pseudomonas-A4	7,030,546	Chr	51.5x	SAMN59779561
	313,044	pQ-KAB	44.3x	NA
Pseudomonas-A8	6,972,108	Chr	37.8x	SAMN59779562
	313,044	pQ-KAB	35.5x	NA
Pseudomonas-A10	6,951,306	Chr	35.4x	SAMN59779563
Pseudomonas-A11	6,951,312	Chr	45.4x	SAMN59779564
Pseudomonas-B2	7,481,942	Chr	64.0x	SAMN59779565
	313,044	pQ-KAB	65.3x	NA
	161,148	Unknown	63.2x	NA
Pseudomonas-B5	6,851,777	Chr	93.3x	SAMN59779566
	313,044	pQ-KAB	94.9x	NA
Pseudomonas-B7	7,014,230	Chr	43.7x	SAMN59779567
	313,044	pQ-KAB	46.3x	NA
Pseudomonas-B8	7,404,182	Chr	100.7x	SAMN59779568
	313,044	pQ-KAB	79.4x	NA
Pseudomonas-C4	7,058,632	Chr	100.3x	SAMN59779569
	313,044	pQ-KAB	83.5x	NA
	51,526	Unknown	277.0x	NA
Pseudomonas-C5	6,899,057	Chr	83.1x	SAMN59779570
	313,044	pQ-KAB	90.7x	NA
Pseudomonas-C9	7,127,274	Chr	81.0x	SAMN59779571
	313,044	pQ-KAB	100.1x	NA

Pseudomonas-C12	6,851,778	Chr	99.5x	SAMN59779572
	313,044	pQ-KAB	108.1x	NA
Pseudomonas-D5	5,627,944	Chr	100.5x	SAMN59779573
	313,044	pQ-KAB	86.4x	NA
Pseudomonas-D7	6,951,321	Chr	58.4x	SAMN59779574
Pseudomonas-D11	7,156,130	Chr	100.2x	SAMN59779575
	313,044	pQ-KAB	92.1x	NA
Pseudomonas-E5	7,246,751	Chr	53.2x	SAMN59779576
	313,044	pQ-KAB	64.0x	NA
Pseudomonas-E12	6,983,138	Chr	100.0x	SAMN59779577
	313,045	pQ-KAB	96.4x	NA
Pseudomonas-F3	6,877,824	Chr	100.1x	SAMN59779578
	313,044	pQ-KAB	100.8x	NA
Pseudomonas-F4	7,246,756	Chr	100.1x	SAMN59779579
	313,045	pQ-KAB	94.3x	NA
Pseudomonas-F6	6,936,404	Chr	100.2x	SAMN59779580
	313,045	pQ-KAB	92.1x	NA

Supplementary Table 3. Naming convention of non-*Pseudomonas* isolates based on their well placement. This naming is used in **Supp Fig 2**.

Well	Taxonomy	Well	Taxonomy
A1	*Microbacterium_D9*	C9	*Xanthomonas_D5*
A2	*Chitinophaga_E11*	C10	*Microbacterium_D6*
A3	*Dyella_F1*	C11	*Pandoraea_E1*
A4	*Acinetobacter_G1*	C12	*Chitinophaga_E6*
A5	*Parapusillimonas_H6*	D1	*Chitinophaga_F10*
A6	*Bacillus_H11*	D2	*Brevundimonas_E10*
A7	*Chitinophaga_H12*	D3	*Chromobacterium_F9*
A8	*Rhodanobacter_A1*	D4	*Aerosticca_F5*
A9	*Rhodanobacter_H7*	D5	*Youhaiella_F6*
A10	*Arthrobacter_A4*	D6	*Brucella_F12*
A11	*Microbacterium_A7*	D7	*Brucella_G1*
A12	*Bordetella_A10*	D8	*Nocardia_F7*
B1	*Castellaniella_B1*	D9	*Pandoraea_G6*
B2	*Dyella_B3*	D10	*Rhodanobacter_F6*
B3	*Bordetella_B4*	D11	*Brucella_G11*
B4	*Castellaniella_B5*	D12	*Pandoraea_G12*
B5	*Microbacterium_B6*	E1	*Luteibacter_E12*
B6	*Brevibacillus_H11*	E2	*Brucella_A2*
B7	*Ochrobactrum_B10*	E3	*Shinella_A5*
B8	*Rhizobium_B11*	E4	*Castellaniella_E6*
B9	*Chitinophaga_C1*	E5	*Pandoraea_A11*
B10	*Luteibacter_C2*	E6	*Pseudarthrobacter_B1*
B11	*Rhodanobacter_C3*	E7	*Pinirhizobacter_B4*
B12	*Castellaniella_C4*	E8	*Treponema_C2*
C1	*Pusillimonas_H12*	E9	*Glutamicibacter_C6*
C2	*Chitinophaga_C7*	E10	*Paeniglutamicibacter_E4*
C3	*Castellaniella_C8*	E11	*Rhodococcus_C9*
C4	*Paenibacillus_C9*	E12	*Candidimonas_C10*
C5	*Rhodanobacter_C10*	F1	*Xanthomonas_E2*
C6	*Paradevosia_C12*	F2	*Castellaniella_D4*
C7	*Bordetella_D2*	F3	*Castellaniella_D6*
C8	*Rhodanobacter_D3*	F4	*Microbacterium_D1*
